# Dual-timing PSA as a biomarker for patients with salvage intensity modulated radiation therapy for biochemical failure after radical prostatectomy

**DOI:** 10.18632/oncotarget.10000

**Published:** 2016-06-14

**Authors:** Yu-Jen Wang, Chao-Yuan Huang, Wei-Hsien Hou, Chia-Chun Wang, Keng-Hsueh Lan, Hong-Jen Yu, Ming-Kuen Lai, Shihh-Ping Liu, Yeong-Shau Pu, Jason Chia-Hsien Cheng

**Affiliations:** ^1^ Department of Radiation Oncology, Shuang Ho Hospital, Taipei Medical University, New Taipei City, Taiwan; ^2^ Division of Radiation Oncology, Department of Oncology, National Taiwan University College of Medicine and Hospital, Taipei, Taiwan; ^3^ Department of Urology, National Taiwan University College of Medicine and Hospital, Taipei, Taiwan; ^4^ Graduate Institute of Oncology, National Taiwan University College of Medicine, Taipei, Taiwan; ^5^ Graduate Institute of Clinical Medicine, National Taiwan University College of Medicine, Taipei, Taiwan

**Keywords:** prostate cancer, radical prostatectomy, biochemical failure, intensity modulated radiation therapy

## Abstract

We investigated the outcomes and the associated clinical-pathological factors in patients with prostate cancer (PCa) undergoing salvage intensity modulated radiation therapy (IMRT) for post-radical-prostatectomy (RP) biochemical failure. We report clinical outcomes of post-RP salvage IMRT, and describe chronic toxicity in these patients.

Fifty patients with PCa underwent post-RP salvage IMRT. The median dose of IMRT was 70 Gy to the prostatic and seminal vesicle bed. Clinical-pathological and toxicity information were collected. The prostate cancer-specific survival (PCSS), disease-free survival (DFS), and biochemical-failure-free survival (BFFS) were calculated. Prognostic factors were analyzed for their association with disease control.

The median follow-up time was 74 months. The 5-year PCSS, DFS, and BFFS after salvage IMRT were 95%, 88%, and 60%, respectively. Two patients (4%) experienced late gastrointestinal toxicity ≥ grade 3, and 5 patients (10%) had late genitourinary toxicity ≥ grade 3. On multivariate analysis, post-RP prostate-specific antigen (PSA) nadir ≤0.1 ng/ml (*P*=0.018) and PSA ≤0.5 ng/ml at salvage IMRT (*P*=0.016) were independent factors predicting better BFFS. Patients with both post-RP PSA nadir ≤0.1 ng/ml and PSA ≤0.5 ng/ml at salvage IMRT had a 5-year BFFS of 83% as compared with 43% in other patients (*P*=0.001).

In conclusion, with hormonal therapy in most PCa patients, the addition of salvage IMRT for post-RP biochemical failure can achieve a good outcome with low toxicity. Patients with a post-RP PSA nadir ≤0.1 ng/ml and PSA ≤0.5 ng/ml at salvage IMRT could benefit the most from salvage IMRT.

## INTRODUCTION

Although prostate cancer is the number one cancer diagnosed in men in the western world, its incidence in Asia is much lower. Rates in Asian countries are up to 60 times less than those reported by the US, and the incidence between Asian countries also varies significantly [1, 2]. In Taiwan, a total of 4,740 patients were diagnosed with prostate cancer in 2012, and the crude cancer incidence rate was 40.61 in 100,000 men [3]. Radical prostatectomy (RP) is a standard treatment for patients with clinically localized PCa and life expectancy >10 years [4]. Even with significant advances in surgical techniques including laparoscopic procedures and robotic surgery, approximately 15–25% of patients who have undergone RP for localized PCa have cancer recurrence [5, 6]. The recurrence manifests initially as an increasing level of serum prostate-specific antigen (PSA), described as biochemical failure [7, 8]. Extracapsular extension, seminal vesicle invasion, and close/positive margin RP were considered to be the characteristics of high-risk groups [9, 10].

Salvage treatment options for post-RP biochemical failure include radiation therapy (RT), hormone therapy, cryotherapy, and high-intensity focused ultrasound (HIFU) [11]. A retrospective study revealed that hormone therapy alone for post-RP biochemical failure only delayed clinical metastasis in patients with a high Gleason score, but had no impact on PCa-specific mortality [12]. In contrast, salvage HIFU and cryotherapy can be used only to treat biopsy-confirmed gross local recurrence. With these treatments, however, the 5-year biochemical-failure-free survival is only around 50% [13, 14].

RT has advanced dramatically in the past few decades, especially with intensity-modulated RT (IMRT) for dose escalation. Definitive IMRT in localized PCa results in lower acute and late toxicities compared with conventional conformal RT techniques [15], and the European Organisation for Research and Treatment of Cancer (EORTC) Radiation Oncology Group guidelines recommend IMRT for primary radiation treatment in patients with PCa [16].

The outcome and toxicity of salvage RT using IMRT technique in PCa patients with post-RP biochemical failure have not been well studied. In this study, we investigated the clinical outcomes of post-RP patients undergoing salvage IMRT for biochemical failure, and analyzed the prognostic factors for the subgroup with the greatest benefit from salvage IMRT.

## RESULTS

### Patient characteristics

The median age at RP was 65 years (range: 49–77), and at salvage IMRT, 67 years (range: 50–84). Of the 50 patients, 18 had initial clinical T1 (36%), 28 had T2 (56%), and four had T3 stage disease (8%). At the time of RP, patients were distributed as follows according to pathological stage: two patients had stage T1 disease (4%), 22 had stage T2 (44%), 25 had stage T3 (50%) and one had stage T4 (2%). The median pre-RP PSA level was 12 ng/ml (range: 2–74). Ten patients (20%) had a Gleason score of 6, 24 (48%) had a score of 7, four (8%) had a score of 8, and 12 (24%) had a score of 9. Four patients (8%) were classified as low-risk, 19 patients (38%) as intermediate-risk, and 27 (54%) patients as high-risk groups based on National Comprehensive Cancer Network (NCCN) risk group stratification. The median PSADT was 4 months (range: 1–54), and median PSAV was 0.4 ng/ml/year (range: 0.1–9.5). All enrolled patients tolerated IMRT well and completed a full course of radiotherapy within 8 weeks. Their baseline characteristics are shown in Table 1.

**Table:** Clinical characteristics of 50 post-prostatectomy localized prostate cancer patients with biochemical failure undergoing salvage intensity modulated radiation therapy (IMRT)

Variable	Patient number	Percent
Total	50	100
Age at IMRT		
< 65	15	30
65-75	28	56
>75	7	14
Initial clinical T stage before RP		
T1	18	36
T2	28	56
T3	4	8
Pathological T stage on RP		
T1	2	4
T2	22	44
T3	25	50
T4	1	2
Initial Gleason score		
6	10	20
7	24	48
8	4	8
9	12	24
PSA before RP		
<10 ng/ml	19	38
10-20 ng/ml	16	32
>20 ng/ml	15	30
Surgical type		
Open RP	38	76
Laparoscopic RP	12	24
PSA doubling time		
<3 months	21	42
3-6 months	14	28
6-12 months	10	20
≥12 months	5	10
Nadir PSA after RP		
<0.1 ng/ml	16	32
0.1-0.2 ng/ml	16	32
0.2-0.5 ng/ml	12	24
>0.5 ng/ml	6	12
PSA velocity		
0.1-0.2 ng/ml/year	11	22
0.2-0.5 ng/ml/year	18	36
0.5-1.0 ng/ml/year	9	18
>1.0 ng/ml/year	12	24
PSA before salvage IMRT		
<0.2 ng/ml	12	24
0.2-0.5 ng/ml	15	30
>0.5 ng/ml	23	46
IMRT dose		
60-63.9 Gy	11	22
64-67.9 Gy	12	24
68-69.9 Gy	1	2
70-74 Gy	26	52
ADT at biochemical failure		
Yes	36	72
No	14	28
ADT duration		
≤ 6 months	11	22
6-12 months	5	10
12-24 months	14	28
24-36 months	6	12

Abbreviations: ADT: androgen deprivation therapy; PSA: prostate specific antigen; RP: radical prostatectomy

### Outcome

**Figure 1:**
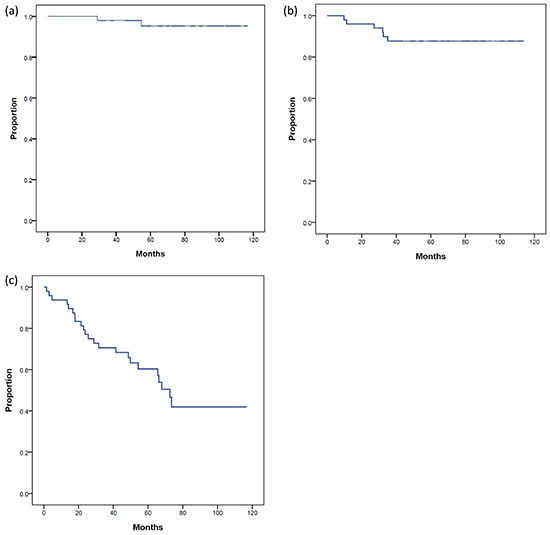
**(a).** prostate cancer specific survival (PCSS), **(b).** disease-free survival (DFS), and **(c).** biochemical failure-free survival (BFFS) of post-radical prostatectomy (RP) patients undergoing salvage intensity modulated radiation therapy for post-RP biochemical failure.

The median follow-up time after salvage IMRT was 74 months (range: 32–116 months). The 5-year PCSS, DFS, and BFFS of these patients after salvage IMRT were 95%, 88%, and 60%, respectively (Figure 1). During follow-up, four patients died, four patients experienced distant metastasis, and two patients had both local recurrence and distant metastasis. The most common metastatic sites were bone (n=4) and lymph node(s) (n=2, both were para-aortic lymph nodes). In total, 25 patients experienced biochemical failure after salvage IMRT. The median BFFS time was 70 months (range: 2-117 months).

### Adverse effects

The majority of patients had grade 0 or 1 acute genitourinary (GU) or gastrointestinal (GI) toxicity when undergoing IMRT (Table 2). After the median 6-year follow-up, one patient (2%) experienced grade 2 toxicity, and two patients (4%) had grade 3 late GI toxicity. Five patients had urinary incontinence requiring absorbent pads before salvage IMRT. Among them, three patients experienced grade 3 late GU toxicity, and two patients continued to have grade 2 GU morbidity. In total, five patients (10%) experienced grade 2 toxicity, and five patients (10%) had grade 3 late GU toxicity. The only GU toxicity other than incontinence was hematuria. There was no change in the post-IMRT status of erectile dysfunction and urinary frequency when compared with post-RP status. No patient had urinary obstruction, urgency, dysuria, or diarrhea after IMRT. The mean volume fraction of rectum that received more than 60 Gy (V60) was 15% (range: 6%-21%). The mean V60 of bladder was 17% (range: 6%-38%). No statistically significant difference in V60 of rectum and bladder was shown between patients with and without grade 2 or more severe GI or GU toxicity.

**Table 2:** Acute and chronic gastrointestinal (GI) and genitourinary (GU) toxicity of post-radical prostatectomy patients who underwent salvage intensity modulated radiation therapy

GI toxicity	Grade 0	Grade 1	Grade 2	Grade 3	Grade 4
Acute	25 (50%)	20 (40%)	5 (10%)	0	0
Late	42 (84%)	5 (10%)	1 (2%)	2 (4%)	0
**GU toxicity**	**Grade 0**	**Grade 1**	**Grade 2**	**Grade 3**	**Grade 4**
Acute	31 (62%)	14 (28%)	5 (10%)	0	0
Late	35 (70%)	5 (10%)	5 (10%)	5 (10%)	0

### Prognostic factors

On univariate analysis, Gleason ≥ 8 was the only significant factor associated with PCSS among the factors (p=0.036), including PSA at salvage IMRT, PSA nadir after RP, PSADT, PSAV, pathological T stage, Gleason score, PSA before RP, ADT use and duration at biochemical failure, the interval from RP to biochemical failure, salvage IMRT dose, pre-RP NCCN risk group and surgical margin status on RP, but was not statistically significant on multivariate analysis (Supplementary Table S1). Only the post-RP PSA nadir ≤0.1 ng/ml was significantly associated with better DFS (*P*=0.008) on univariate analysis, but was not statistically significant on multivariate analysis (Supplementary Table S2).

**Figure 2:**
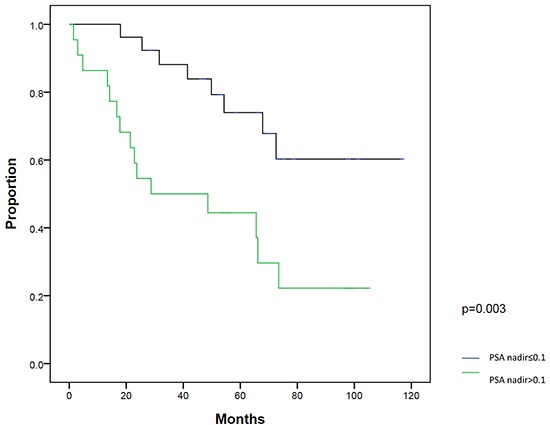
Biochemical failure-free survival (BFFS) between patients with post-radical prostatectomy PSA nadir ≤0.1 ng/ml and >0.1 ng/ml

**Figure 3:**
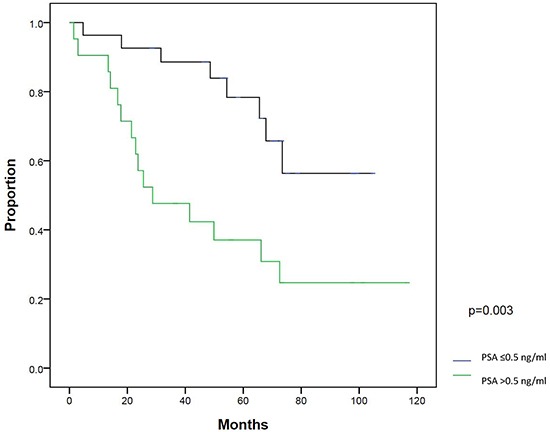
Biochemical failure-free survival (BFFS) between patients with PSA ≤0.5 ng/ml and >0.5 ng/ml at salvage intensity modulated radiation therapy

**Figure 4:**
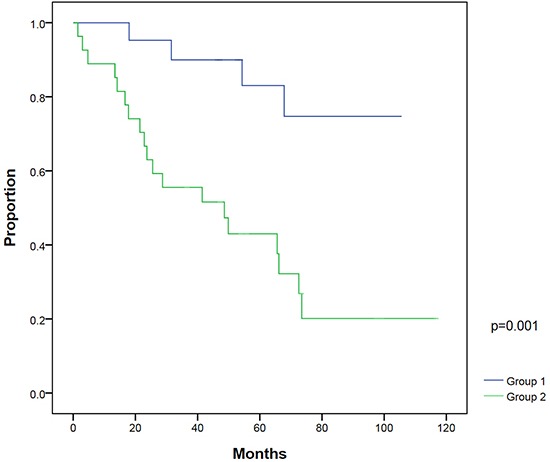
Biochemical failure-free survival between patients with post-radical prostatectomy (RP) PSA nadir ≤0.1 ng/ml and PSA ≤0.5 ng/ml at salvage intensity modulated radiation therapy (IMRT) (group 1) compared with patients with post-RP PSA nadir >0.1 ng/ml and/or PSA at salvage IMRT >0.5 ng/ml (group 2)

On univariate analysis, patients with a post-RP PSA nadir ≤0.1 ng/ml (*P*=0.003) and PSA at salvage IMRT ≤0.5 ng/ml (*P*=0.003) had significantly better BFFS. On multivariate analysis, post-RP PSA nadir ≤0.1 ng/ml (*P*=0.018) and PSA at salvage IMRT ≤0.5 ng/ml (*P*=0.016) were two statistically significant independent factors for BFFS (Table 3). The 5-year BFFS in patients with a post-RP PSA nadir ≤0.1 ng/ml was 74.0%, and for those with a nadir of >0.1 ng/ml, the 5-year BFFS was 44.4% (Figure 2). The 5-year BFFS in patients with PSA at salvage IMRT ≤0.5 ng/ml was 78.3%, and for those with PSA at salvage IMRT >0.5 ng/ml the 5-year BFFS was 37.0% (Figure 3). The favorable-group patients with post-RP PSA nadir ≤0.1 ng/ml and PSA at salvage IMRT ≤0.5 ng/ml had a 5-year BFFS of 83%, compared with 43% for other patients (Figure 4). We also conducted the tests to check the same risk factors for NCCN low- to intermediate-risk patients (n=23) and high-risk (n=27) patients, respectively. For high-risk patients, univariate analysis showed PSA ≤0.5 ng/ml at salvage IMRT had a better 5-year BFFS. (Supplementary Table S4). For low- to intermediate-risk patients, univariate analysis showed post-RP PSA nadir ≤0.1 ng/ml and PSA at salvage IMRT ≤0.5 ng/ml had a better 5-year BFFS (Supplementary Table S5).

**Table 3:** Univariate and multivariate analyses of the prognostic factors on biochemical failure-free survival (BFFS) of post-radical prostatectomy (RP) patients with biochemical failure undergoing salvage intensity modulated radiation therapy (IMRT)

Variable	Patient numbers	Five-year BFFS	*p* value	HR (95% CI)	*p* value
PSA at salvage IMRT					
>0.5 ng/ml	22	37.0%	0.003	0.340 (0.141-0.817)	0.016
≤0.5 ng/ml	28	78.3%			
PSA nadir after RP					
>0.1 ng/ml	23	44.4%	0.003	0.346 (0.143-0.832)	0.018
≤0.1 ng/ml	27	74.0%			
PSA doubling time					
≥3months	24	61.7%	0.571		
<3 months	26	59.1%			
PSA velocity					
≤0.5 ng/ml/year	30	54.5%	0.358		
>0.5/ng/ml/year	20	68.4%			
Pathological T stage					
T3-T4	26	58.4%	0.844		
T1-T2	24	63.6%			
Gleason score					
8-10	16	58.3%	0.931		
≤7	34	61.3%			
Initial PSA before RP					
≥20 ng/ml	14	67%	0.831		
<20 ng/ml	36	56%			
Androgen-deprivation therapy use at biochemical failure					
Yes	36	65.5%	0.267		
No	14	50.0%			
Salvage IMRT dose					
<70 Gy	24	67.0%	0.245		
≥70 Gy	26	52.4%			
Surgical margin on RP					
Positive	32	62.3%	0.261		
Negative	18	56.6%			
ADT duration					
≦6 months	18	72.9	0.451		
>6 months	18	61.9			
NCCN Risk group					
Low and intermediate risk	23	63.6	0.844		
High risk	27	58.4			

Abbreviations: ADT: androgen-deprivation therapy; CI: confidence interval; HR: hazard ratio; IMRT: intensity modulated radiation therapy; PSA: prostate specific antigen; RP: radical prostatectomy; NCCN: National Comprehensive Cancer Network

## DISCUSSION

Our study demonstrates that post-RP patients with biochemical failure treated by salvage IMRT and ADT (in 72% of patients) achieved good clinical outcome. Long-term grade 2 GU and GI toxicity was as low as 10% and 4% for patients treated by IMRT with a median dose of 70 Gy, respectively, compared with 21% and 9% in previous studies by two-dimensional RT technique with an average dose of only 64.6 Gy [17]. Patients who had a post-RP PSA nadir below or equal to 0.1 ng/ml and PSA level lower than or equal to 0.5 ng/ml before salvage IMRT had a 5-year BFFS of 83%. Thus, we refer to these patients as the favorable group. Our study may be novel with a high median dose of 70 Gy, uniform contouring, and IMRT exclusively used in PCa patients with post-RP biochemical failure.

Several studies have reported clinical outcomes and prognostic factors for salvage RT for post-RP PCa patients with biochemical failure. Stephenson et al. reported their results from 501 patients receiving traditional RT with a median RT dose of 64.8 Gy. The probability of 6-year progression-free status was 32%. The Gleason score, pre-RT PSA level, surgical margins, PSADT, and seminal vesicle invasion were prognostic variables for a durable response to salvage radiotherapy, and a nomogram was developed [18, 19]. Ying et al. treated 61 patients with a median RT dose of 64.8 Gy, with a 33% rate of 10-year freedom from PSA failure [20]. Goenka et al. showed a 37% 7-year actuarial PSA-relapse-free survival in 285 patients, with 72% of patients receiving an RT dose of more than 70 Gy [21]. Makito et al. showed a 5-year BFFS of 38% after salvage RT (70 Gy) without hormone therapy [22]. Mizowaki et al. reported the results from the Japanese Radiation Oncology Study Group (JROSG) who showed that the 5-year PSA recurrence-free survival and clinical-failure-free survival rates were 50.1% and 90.1%, respectively [23]. Although it is not easy to compare the data between the studies directly, our 5-year BFFS of 60% is not inferior to the rates in the published series.

In addition, the radiation dose and long-term treatment-related toxicity are two important issues for PCa patients with post-RP biochemical failure treated by radiotherapy. IMRT provides an effective protocol for dose escalation as well as reduction of adverse effects. King et al. published the results of a retrospective study comparing the outcomes of 38 patients treated with 60 Gy, and 84 patients with 70 Gy. They demonstrated a significantly improved 5-year BFFS, from 25% to 58%, with the higher doses [24]. In contrast, our data on IMRT dose failed to detect a biochemical difference. Similarly reported by Goenka et al., salvage RT dose ≥70 Gy was not associated with improved biochemical control, but was associated with a borderline benefit in preventing clinical local failure in patients with radiographically visible local disease at salvage RT [21].

The Memorial Sloan-Kettering Cancer Center compared the toxicity profile between three-dimensional RT and IMRT, and found IMRT to be independently associated with a reduction of GI toxicity ≥ grade 2 compared with three-dimensional RT (1.9% vs. 10.2%). Notably, their 5-year rate of GU toxicity ≥ grade 2 in patients treated with IMRT was 16.8% [25]. The JROSG reported that late GI and GU adverse events ≥ grade 2 were 4.3% and 16.1%, respectively [23]. It is noteworthy that our data with even longer follow-up time (median 74 months) showed comparable rates of GI and GU toxicity ≥ grade 2 in 6% and 20% of patients, respectively. It is of note that our study included patients treated with IMRT, volumetric modulated arc therapy (VMAT), and tomotherapy. Our previous study on the dosimetric comparison between these techniques revealed the better normal tissue sparing by VMAT but no difference in the target coverage [26].

Other studies similarly have found the favorable prognostic factors of post-RP PSA nadir and pre-RT PSA that our series reported. An early study reported that patients with a low PSA level (≤2 ng/ml) at the time of RT had the best outcomes [27]. A study from the Memorial Sloan-Kettering Cancer Center showed that a pre-RT PSA level >0.4 ng/ml was an independent prognostic factor [21]. As for the post-RP PSA nadir, Garg et al. showed that patients with undetectable postoperative PSA had slightly better disease-free survival [27]. Doherty et al. reported that PCa patients with PSA undetectable by ultrasensitive PSA assay after RP had better relapse-free survival [28].

Based on American Urological Association/American Society for Radiation Oncology guidelines, salvage RT is the administration of RT to the prostatic bed and possibly to the surrounding tissues, including lymph nodes, in the patient with a PSA recurrence after surgery but no evidence of distant metastatic disease [29]. In our study, we showed the significant difference in BFFS but not in DFS or PCSS. This is likely related to the limited follow-up time (median 74 months) and the comorbidity from the old-age PCa patients. Similarly, Goenka et al. [21] and Makito et al. [22] did not show the prognostic factor of DFS or PCSS.

In this study, 72% of patients had ADT during or after radiotherapy, mainly those patients with risk factors associated with high risk. Although the use of ADT was not a significant factor for disease control, this heterogeneity might bias the analysis. ADT, alone or combined with other treatments, undoubtedly has been a viable option for treating PCa patients with post-RP biochemical failure. Given that less than 30% patients received ADT in previous similar studies, our study with ADT use in 72% of patients might have the outcome difference partly from ADT. The same situation was found in most published studies, with various benefits reported (Supplementary Table S3). Some retrospective studies showed a PSA-relapse-free survival advantage compared with RT alone [21, 30], while others failed to demonstrate benefit [19, 20]. Heterogeneous risk categories in patient selection for ADT use, the duration of ADT, and the endpoint difference (clinical or biochemical) from these retrospective studies confounds the interpretation of benefit in disease control. In the PSA era, notably, the phase III clinical trial (RTOG-9601), comparing RT (64.8 Gy) with RT plus 2 years of high-dose bicalutamide (150 mg per day) showed that the addition of ADT during and after RT significantly lengthened the time to PSA progression, and reduced the incidence of distant metastasis [31]. More evidence from the prospective studies is needed to elucidate the role of ADT in patients with biochemical failure undergoing IMRT.

Because of its retrospective study design, our series inevitably has some limitations. A relatively small sample size from a single center made selection bias difficult to avoid. Furthermore, inhomogeneous use of hormones in this study could confound the findings. Of note, some molecular markers such as CD117+ cell levels and miRNAs (miR-103, 125b, and 222) expression have been proposed for the prediction of biochemical failure for patients after RP and decide timing of salvage treatment [32, 33]. The prospective multi-center study will be needed to confirm the results.

In conclusion, post-RP salvage IMRT achieved satisfactory clinical outcomes and acceptable toxicity in post-RP PCa patients with biochemical failure. The patients who had a post-RP PSA nadir ≤ 0.1 ng/ml, and PSA level ≤ 0.5 ng/ml at salvage IMRT had the best BFFS.

## MATERIALS AND METHODS

### Patients

From 2004 to 2012, 69 patients with PCa underwent post-RP IMRT at National Taiwan University Hospital. Inclusion criteria for this study were PCa (adenocarcinoma) in patients who underwent post-RP IMRT for biochemical failure with no detectable gross recurrence by digital rectal examination and computed tomography or magnetic resonance imaging of the pelvis. Sixteen patients who underwent post-RP adjuvant RT, and three patients with pathological positive pelvic lymph node(s) on RP were excluded. A total of 50 patients were enrolled in this series. Medical records were reviewed for the relevant clinical-pathological factors and treatment-related toxicity information. The highest Gleason score from either the prostate biopsy or the RP specimen represented the tumor grade. The 7th American Joint Committee on Cancer TNM classification was used for staging the disease at the time of RP. The definition of post-RP biochemical failure is a PSA level of >0.20 ng/ml detected on two consecutive measurements with the interval of at least 3 months [11]. PSA doubling time (PSADT) and PSA velocity (PSAV) between the post-RP PSA nadir and the initiation of salvage RT were calculated using at least two PSA measurements with a 3-month interval and log calculations on the website of the Memorial Sloan Kettering Cancer Center (http://nomograms.mskcc.org/Prostate/PsaDoublingTime.aspx). The study was conducted in compliance with the protocol and in accordance with the provisions of the Declaration of Helsinki (2010), and was approved by the institutional review board.

### Treatments

Among the 50 patients with post-RP biochemical failure, 38 patients underwent open RP, and 12 patients had laparoscopic RP. All of these patients underwent IMRT, and the median RT dose was 70 Gy (range: 63–74 Gy) by 6-MV (Tomotherapy) or 10-MV photon radiation with 1.8 or 2.0 Gy per fraction per day. For IMRT, the clinical target volume (CTV) was prostatic and seminal vesicle bed plus periprostatic tissues as the EORTC guidelines for target volume definition in post-operative radiotherapy for PCa [34]. Planning target volume (PTV) expansions were 6 mm posteriorly (rectum), 6 mm inferiorly, 10 mm anteriorly, bilaterally, and superiorly from CTV. Patients were treated in a prone position, and a 60-ml air-filled endorectal balloon was placed in each fraction of IMRT to immobilize the prostatic bed and reduce rectal toxicity. The treatment goal was 100% of prescribed radiation dose covering >95% of the PTV, with the maximum not exceeding 110%. Routine on-board cone-beam computed tomography was used to verify target positions. In the study period the IMRT techniques evolved from step-and-shoot IMRT (39 patients), Tomotherapy (2 patients), to volumetric modulated radiation therapy (9 patients), and the prescribed dose was increased with the advancement of technology and better organ sparing. Androgen-deprivation therapy (ADT) was given in 36 patients, based mainly on factors associated with high risk, including high PSA nadir after RP, high Gleason grade, and short PSADT. For patients treated with ADT and IMRT, ADT was administered as a neoadjuvant more than two months prior to RT, and was continued concurrently with RT. Alternatively, maintenance ADT was administered concurrently with IMRT and was continued after IMRT for 12 months. Patients typically received gonadotropin-releasing hormone (GnRH) agonist as monotherapy. An oral anti-androgen was usually initiated at the start of GnRH agonist therapy to prevent a rebound surge of androgen.

### Follow-up

Patients were followed at the out-patient clinic every 3 months in the first 3 years and every 6 months after 3 years with history taking, digital rectal examination, and PSA. Follow-up duration, survival time, and event time were calculated from the start of salvage IMRT. Kaplan-Meier analysis was performed to determine prostate cancer-specific survival (PCSS), disease-free survival (DFS), and biochemical-failure-free survival (BFFS) rates. DFS was defined as survival in the absence of clinical local recurrence or metastasis. Post-IMRT biochemical failure is defined as the detection of a PSA level of >0.20 ng/ml by two consecutive measurements [11]. Treatment-related toxicities were determined using Common Toxicity Criteria v.4.0.

### Statistics

Descriptive analysis was performed by calculating ranges, means, medians, and standard deviations. Continuous variables were compared with a two-sided unpaired *t*-test. Chi-square and Fisher exact test were used for contingency table analysis. The log-rank test was used to determine prognostic factors affecting survival. All prognostic variables found to be significant or borderline significant in univariate analysis were included in multivariate analysis using the Cox proportional hazards regression model. Statistical significance was defined as *P*<0.05. All statistics were done with PASW Statistics 18 (IBM Corp., Armonk, NY, USA).

## SUPPLEMENTARY TABLES










