# Supplementary material for: Dual-timing PSA as a biomarker for patients with salvage intensity modulated radiation therapy for biochemical failure after radical prostatectomy
Source: Oncotarget. 2016 Jun 14;7(28):44224–35. doi: 10.18632/oncotarget.10000 (PMC5190091; doi:10.18632/oncotarget.10000)
Supplement: Supplementary file 1 [file oncotarget-07-44224-s001.pdf]

Dual-timing PSA as a biomarker for patients with salvage intensity modulated radiation therapy for biochemical failure after radical prostatectomy

SUPPLEMENTARY TABLES

Supplementary Table S1: Univariate analysis of the prognostic factors on prostate cancer specific survival (PCSS) of post-radical prostatectomy (RP) patients with biochemical failure undergoing salvage intensity modulated radiation therapy (IMRT).

See Supplementary File 1

Supplementary Table S2: Univariate and multivariate analyses of the prognostic factors on disease-free survival (DFS) of post-radical prostatectomy (RP) patients with biochemical failure undergoing salvage intensity modulated radiation therapy (IMRT).

See Supplementary File 2

Supplementary Table S3: Various benefits of androgen-deprivation therapy (ADT) use in patients who underwent salvage radiation therapy

Reference	Patient numbers	ADT use percentage	ADT duration	Outcome	*p* value
Stephenson et al., 2004 [19]	488	17%	Median 3 months	No benefit	0.77
Goenka et al., 2012 [21]	280	31%	---	Better biochemical free survival	0.003
Jackson et al., 2015 [30]	686	21%	Median 12 months	Better biochemical free survival	0.003
Ying et al., 2015 [20]	61	22%	---	No benefit	0.62

Supplementary Table S4: Univariate and multivariate analyses of the prognostic factors on biochemical failure-free survival (BFFS) of post-radical prostatectomy (RP) high-risk patients (n=27) with biochemical failure undergoing salvage intensity modulated radiation therapy (IMRT).

See Supplementary File 3

Supplementary Table S5: Univariate and multivariate analyses of the prognostic factors on biochemical failure-free survival (BFFS) of post-radical prostatectomy (RP) low- to intermediate-risk patients (n=27) with biochemical failure undergoing salvage intensity modulated radiation therapy (IMRT).

See Supplementary File 4
