# Supplementary material for: Dual-timing PSA as a biomarker for patients with salvage intensity modulated radiation therapy for biochemical failure after radical prostatectomy
Source: Oncotarget. 2016 Jun 14;7(28):44224–35. doi: 10.18632/oncotarget.10000 (PMC5190091; doi:10.18632/oncotarget.10000)
Supplement: Supplementary file 2 [file oncotarget-07-44224-s002.docx]

Supplementary table 1 Univariate analysis of the prognostic factors on prostate cancer specific survival (PCSS) of post-radical prostatectomy (RP) patients with biochemical failure undergoing salvage intensity modulated radiation therapy (IMRT)

| Variable | 5-year PCSS | | | *p* value | HR (95% CI) | *p* value |
| --- | --- | --- | --- | --- | --- | --- |
| PSA at salvage IMRT | |  | |  |  |  |
| >0.5 ng/ml | | 94.7% | | 0.986 |  |  |
| ≤0.5 ng/ml | | 96.3% | |  |  |  |
| PSA nadir after RP | |  | |  |  |  |
| >0.1 ng/ml | | 90.2% | | 0.138 |  |  |
| ≤0.1 ng/ml | | 100% | |  |  |  |
| PSA doubling time | |  | |  |  |  |
| ≥3 months | | 100% | | 0.211 |  |  |
| <3 months | | 91.7% | |  |  | |
| PSA velocity | |  | |  |  | |
| ≤0.5 ng/ml/year | | 100% | | 0.095 | 0.462(0.001-6841012.016) | 0.462 |
| >0.5/ng/ml/year | | 88.9% | |  |  |  |
| Pathological T stage | |  | |  |  |  |
| T3-T4 | | 91.2% | | 0.186 |  |  |
| T1-T2 | | 100% | |  |  | |
| Gleason score | |  | |  |  |  |
| 8-10 | | 85.2% | | 0.036 | 160.519(0.000-8.277E7) | 0.449 |
| ≤7 | | 100% | |  |  | |
| Initial PSA before RP | |  | |  |  |  |
| ≥20 ng/ml | | 90.9% | | 0.555 |  |  |
| <20 ng/ml | | 97.1% | |  |  | |
| Androgen-deprivation therapy use at biochemical failure | | |  |  |  |  |
| Yes | | 97.1% | | 0.601 |  |  |
| No | | 91.7% | |  |  | |
| Salvage IMRT dose | |  | |  |  | |
| <70 Gy | | 96.0% | | 0.874 |  | |
| ≥70 Gy | | 95.5% | |  |  | |
| Surgical margin on RP | |  | |  |  | |
| Positive | | 96.9% | | 0.714 |  | |
| Negative | | 92.9% | |  |  | |
| ADT duration | |  | |  |  | |
| ≦6 months | | 94.4% | | 0.977 |  | |
| > 6 months | | 95.8% | |  |  | |
