# Supplementary material for: Dual-timing PSA as a biomarker for patients with salvage intensity modulated radiation therapy for biochemical failure after radical prostatectomy
Source: Oncotarget. 2016 Jun 14;7(28):44224–35. doi: 10.18632/oncotarget.10000 (PMC5190091; doi:10.18632/oncotarget.10000)
Supplement: Supplementary file 4 [file oncotarget-07-44224-s004.docx]

Supplementary table 4 Univariate and multivariate analyses of the prognostic factors on biochemical failure-free survival (BFFS) of post-radical prostatectomy (RP) high-risk patients (n=27) with biochemical failure undergoing salvage intensity modulated radiation therapy (IMRT)

| Variable | Patient numbers | | | Five-year BFFS | *p* value | HR (95% CI) | *p* value |
| --- | --- | --- | --- | --- | --- | --- | --- |
| PSA at salvage IMRT | |  | |  |  |  |  |
| >0.5 ng/ml | | 10 | | 33.3% | 0.036 | 0.294(0.096-0.906) | 0.033 |
| ≤0.5 ng/ml | | 17 | | 72.1% |  |  |  |
| PSA nadir after RP | |  | |  |  |  |  |
| >0.1 ng/ml | | 11 | | 43.6% | 0.105 | 0.367(0.119-1.136) | 0.082 |
| ≤0.1 ng/ml | | 16 | | 69.2% |  |  |  |
| PSA doubling time | |  | |  |  |  |  |
| ≥3months | | 10 | | 37.0% | 0.223 |  |  |
| <3 months | | 17 | | 64.2% |  |  | |
| PSA velocity | |  | |  |  |  | |
| ≤0.5 ng/ml/year | | 14 | | 52.7% | 0.206 |  | |
| >0.5/ng/ml/year | | 13 | | 69.2% |  |  | |
| Gleason score | |  | |  |  |  |  |
| 8-10 | | 16 | | 70.1% | 0.258 |  |  |
| ≤7 | | 11 | | 51.3% |  |  | |
| Initial PSA before RP | |  | |  |  |  |  |
| ≥20 ng/ml | | 14 | | 60.6% | 0.484 |  |  |
| <20 ng/ml | | 13 | | 58.7% |  |  | |
| Androgen-deprivation therapy use at biochemical failure | |  | |  |  |  |  |
| Yes | | 19 | | 61.8% | 0.678 |  |  |
| No | | 8 | | 51.9% |  |  | |
| Salvage IMRT dose | | |  |  |  |  |  |
| <70 Gy | | 11 | | 59.7% | 0.967 |  |  |
| ≥70 Gy | | 16 | | 57.0% |  |  | |
| Surgical margin on RP | |  | |  |  |  | |
| Positive | | 21 | | 62.3% | 0.129 |  | |
| Negative | | 6 | | 50.0% |  |  | |
| ADT duration | |  | |  |  |  | |
| ≦6 months | | 9 | | 70.8 | 0.834 |  | |
| > 6 months | | 9 | | 61.3 |  |  | |

ADT: androgen-deprivation therapy; CI: confidence interval; HR: hazard ratio; IMRT: intensity modulated radiation therapy; PSA: prostate specific antigen; RP: radical prostatectomy
