# Supplementary material for: Dual-timing PSA as a biomarker for patients with salvage intensity modulated radiation therapy for biochemical failure after radical prostatectomy
Source: Oncotarget. 2016 Jun 14;7(28):44224–35. doi: 10.18632/oncotarget.10000 (PMC5190091; doi:10.18632/oncotarget.10000)
Supplement: Supplementary file 5 [file oncotarget-07-44224-s005.docx]

Supplementary table 5 Univariate and multivariate analyses of the prognostic factors on biochemical failure-free survival (BFFS) of post-radical prostatectomy (RP) low- to intermediate-risk patients (n=27) with biochemical failure undergoing salvage intensity modulated radiation therapy (IMRT)

| Variable | Patient numbers | | | Five-year BFFS | *p* value | HR (95% CI) | *p* value |
| --- | --- | --- | --- | --- | --- | --- | --- |
| PSA at salvage IMRT | |  | |  |  |  |  |
| >0.5 ng/ml | | 12 | | 41.7% | 0.042 | 0.269(0.052-1.392) | 0.245 |
| ≤0.5 ng/ml | | 11 | | 90.0% |  |  |  |
| PSA nadir after RP | |  | |  |  |  |  |
| >0.1 ng/ml | | 12 | | 45.5% | 0.018 | 0.376 (0.072-1.954) | 0.117 |
| ≤0.1 ng/ml | | 11 | | 81.8% |  |  |  |
| PSA doubling time | |  | |  |  |  |  |
| ≥3months | | 14 | | 71.4% | 0.128 |  |  |
| <3 months | | 9 | | 50.0% |  |  | |
| PSA velocity | |  | |  |  |  | |
| ≤0.5 ng/ml/year | | 16 | | 62.5% | 0.626 |  | |
| >0.5/ng/ml/year | | 7 | | 66.7% |  |  | |
| Androgen-deprivation therapy use at biochemical failure | |  | |  |  |  |  |
| Yes | | 17 | | 68.8% | 0.600 |  |  |
| No | | 6 | | 50.0% |  |  | |
| Salvage IMRT dose | |  | |  |  |  |  |
| <70 Gy | | 13 | | 76.9% | 0.144 |  |  |
| ≥70 Gy | | 10 | | 44.4% |  |  | |
| Surgical margin on RP | |  | |  |  |  |  |
| Positive | | 11 | | 61.5% | 0.921 |  |  |
| Negative | | 12 | | 66.7% |  |  | |
| ADT duration | | |  |  |  |  |  |
| ≦6 months | | 9 | | 75.0 | 0.814 |  |  |
| >6 months | | 9 | | 62.5 |  |  | |

ADT: androgen-deprivation therapy; CI: confidence interval; HR: hazard ratio; IMRT: intensity modulated radiation therapy; PSA: prostate specific antigen; RP: radical prostatectomy
